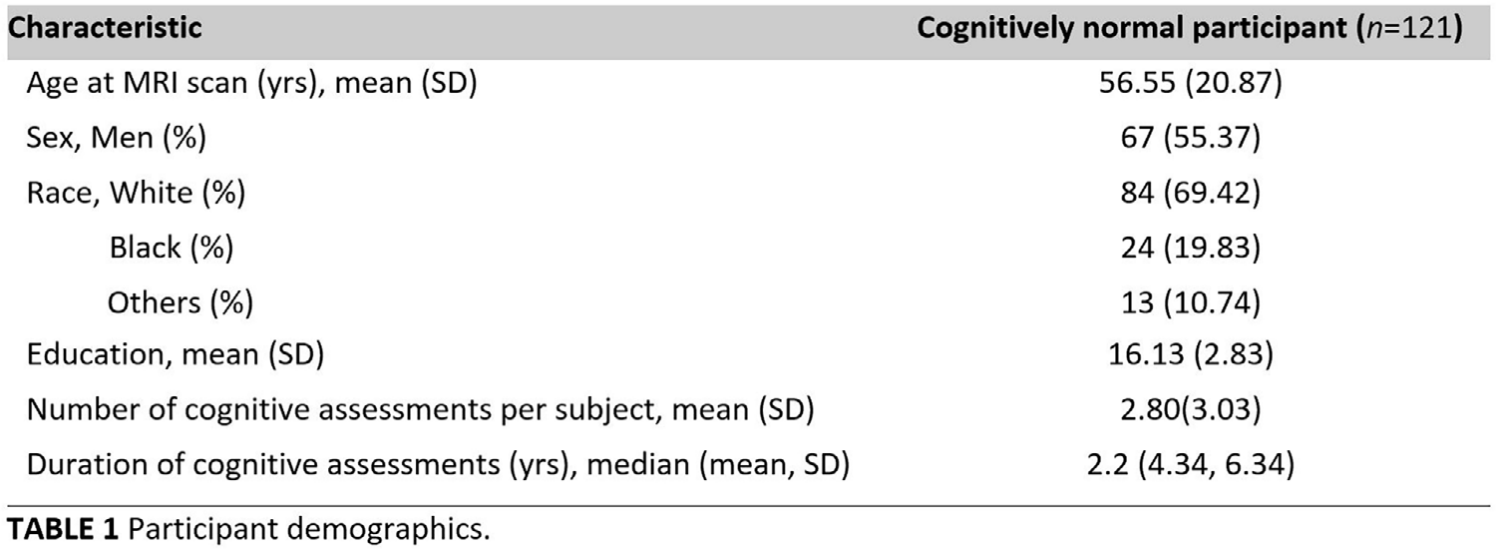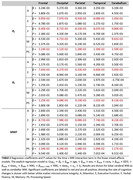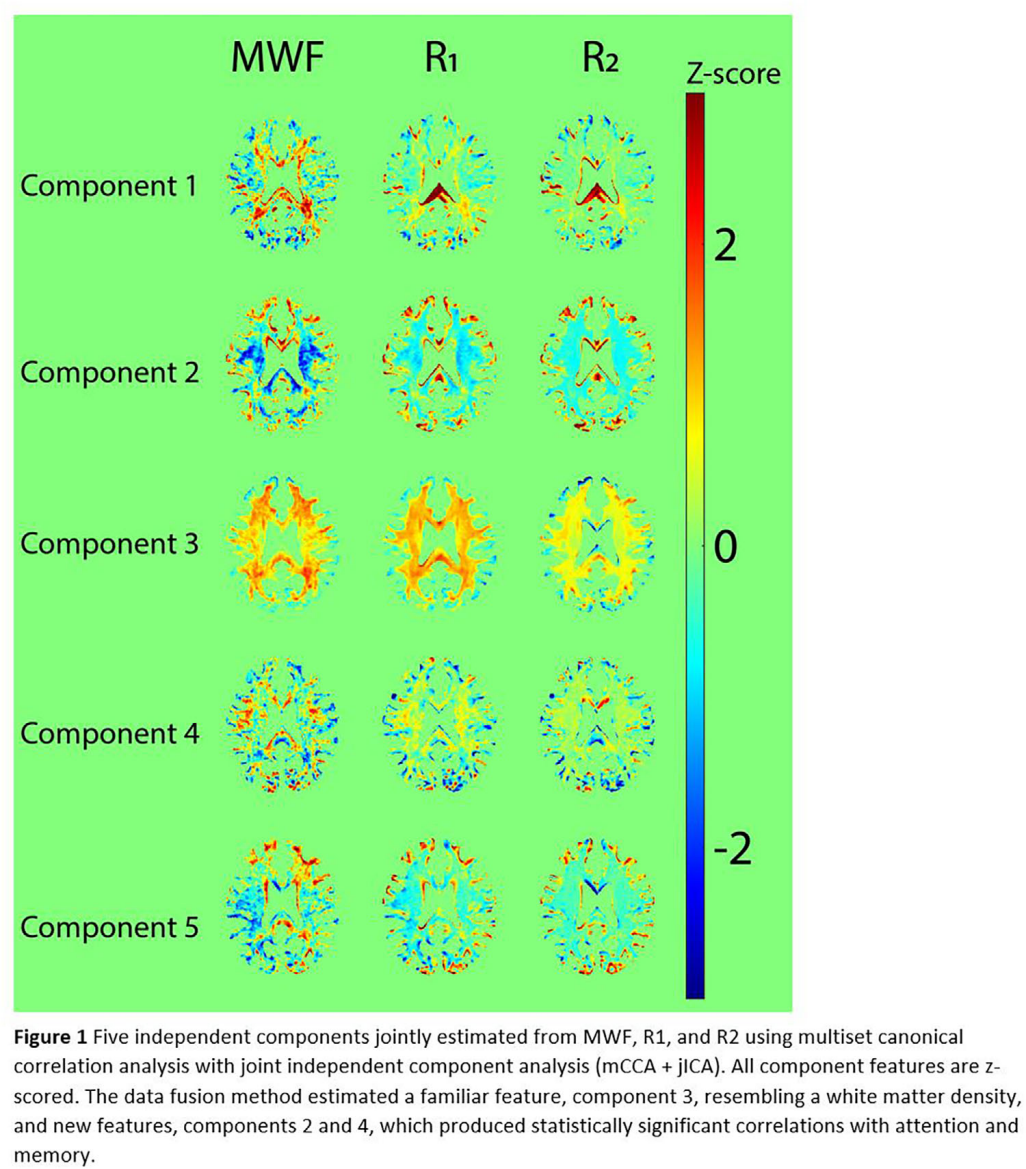# White Matter, Aging, and Cognition: New Insights Using Advanced MR Relaxometry and Symmetric Data‐driven Fusion

**DOI:** 10.1002/alz.093849

**Published:** 2025-01-09

**Authors:** Zhaoyuan Gong, Murat Bilgel, Mary E Faulkner, Jonghyun Bae, John P Laporte, Alex Guo, Mustapha Bouhrara

**Affiliations:** ^1^ National Institute on Aging, Baltimore, MD USA; ^2^ National Institute on Aging, National Institutes of Health, Baltimore, MD USA; ^3^ Laboratory of Clinical Investigation, National Institute on Aging, Intramural Research Program, Baltimore, MD USA

## Abstract

**Background:**

Cognitive decline during normative aging significantly impacts the quality of life, while the rate varies among individuals. MRI studies have highlighted the correlation between cognitive functions and brain macrostructure. However, cerebral microstructural alterations, especially in white matter, may precede macrostructural changes, driving early cognitive decline. Using advanced MR relaxometry, we measured R1 and R2 relaxation rates and Myelin Water Fraction (MWF) to elucidate the relationship between microstructural differences and cognitive decline in aging. Additionally, to leverage the shared information across all MRI metrics, we used advanced data fusion techniques to derive new features in identifying significant relationships with cognition that cannot be seen with a single MRI metric.

**Method:**

121 cognitively unimpaired participants from the BLSA and GESTALT studies underwent cognitive testing. R1, R2, and MWF were calculated using BMC‐mcDESPOT. Multiset canonical correlation analysis with joint independent component analysis (mCCA + jICA) was used to estimate five new white matter integrity features. Linear mixed‐effects models assessed associations between white matter integrity and longitudinal cognitive changes, accounting for age, sex, education, and race.

**Result:**

Lower R1, R2, and MWF values, indicating worse microstructural integrity and low myelin content, were all significantly associated with steeper decline in executive function and processing speed across all regions‐of‐interest (ROIs). Moreover, data fusion yielded five new white matter integrity features (Figure 1). Lower loading coefficients of component 4 were significantly associated with steeper decline in executive function, processing speed and attention. Interestingly, lower loading coefficients of component 2 were significantly associated with steeper decline in verbal fluency and memory. These associations with attention and memory were not observed using any of the single MRI metrics, demonstrating the power and sensitivity of data fusion.

**Conclusion:**

This study establishes a link between white matter microstructural integrity and the rate of cognitive decline in normative aging, suggesting that MRI metrics and their fusion features are not only reflective of microstructural changes but are also predictive of cognitive trajectory. These findings highlight the potential of MR relaxometry in early intervention and contribute to the growing body of evidence supporting the importance of white matter health in cognitive aging.